# Congenital Holoprocencephaly, Hydrocephalus, and Dandy-Walker Malformation Due to Plasminogen Deficiency

**DOI:** 10.7759/cureus.94020

**Published:** 2025-10-07

**Authors:** Nikolaos Antonakopoulos, Panagiotis Perdikaris, Ioanna Styliara, Panagiota Tzela, Georgios Adonakis

**Affiliations:** 1 Department of Obstetrics and Gynecology, School of Health Sciences, University of Patras, Patras, GRC; 2 Department of Obstetrics and Gynecology, University of Patras, School of Medicine, Patras, GRC; 3 Department of Midwifery, School of Health and Care Sciences, University of West Attica, Athens, GRC

**Keywords:** congenital hydrocephalus, dandy walker malformation, holoprocencephaly, plasminogen deficiency, ventriculoperitoneal shunt

## Abstract

Plasminogen deficiency is an ultra-rare congenital condition with only a few reported cases worldwide, causing the formation of pseudo-membranous, fibrin-rich growths on mucosa throughout the body. In this case report, we present the rare case of a newborn presenting with multiple congenital central nervous system malformations due to severe plasminogen deficiency, in an otherwise completely unattended pregnancy. The mother was admitted to the emergency department due to the onset of labor. Upon routine ultrasound examination, marked macrocephaly and hydroanencephaly were observed. The fetus was delivered by selective cesarean section as fetal head dystocia was highly suspected. Unexpectedly, the neonatal examination immediately post-partum did not reveal any neurological deficits. An MRI was conducted, which confirmed the existence of congenital hydrocephalus, as well as holoprocencephaly, Dandy-Walker malformation, and anatomical anomalies of the fourth ventricle. Specialized further laboratory testing pinpointed severe plasminogen deficiency as the underlying condition. The neonate underwent ventriculoperitoneal shunt placement in an attempt to alleviate the increased intracerebral pressure and survived at least to six months of age, at the time this manuscript was written.

## Introduction

Plasminogen deficiency is an ultra-rare congenital disorder characterized by impaired fibrinolysis and the accumulation of fibrin-rich pseudomembranes on mucosal surfaces throughout the body. Fewer than a few hundred cases have been described worldwide, and the clinical spectrum is highly variable, ranging from ligneous conjunctivitis to life-threatening systemic involvement. Neurological manifestations of plasminogen deficiency are exceedingly uncommon, with only isolated reports linking the condition to central nervous system anomalies. Here, we present a rare case of a newborn with multiple congenital brain malformations - including hydrocephalus, holoprosencephaly, and Dandy-Walker malformation - in the setting of severe plasminogen deficiency. Despite its rarity, recognition of plasminogen deficiency is critical, as timely diagnosis and management can alter outcomes and improve quality of life.

## Case presentation

**Figure 1:**
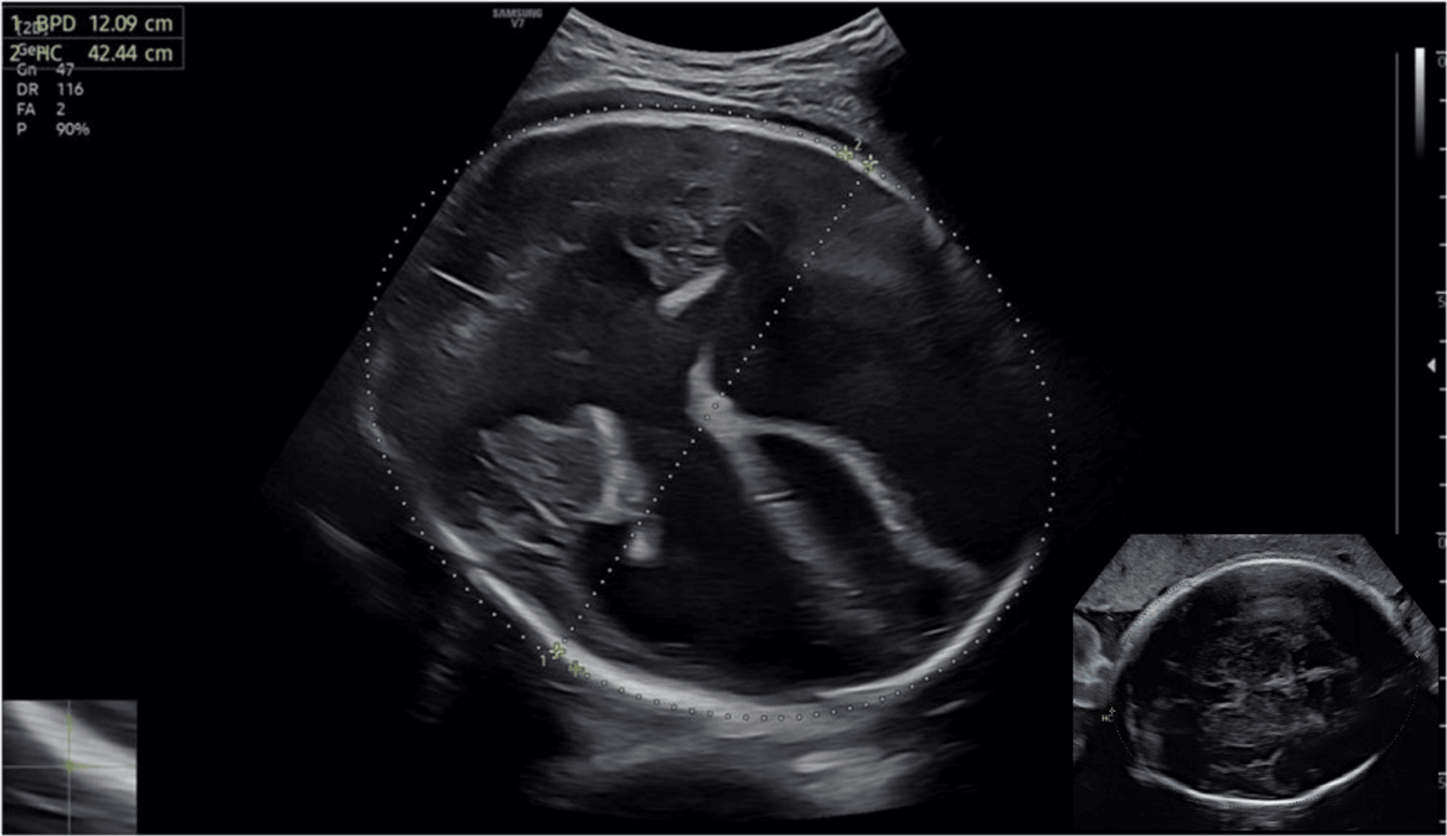
Marked fetal macrocephaly and hydranencephaly. The smaller image on the lower right quadrant shows the normal fetal brain sonographic imaging of the late third trimester.

A 19-year-old pregnant woman presented to the Emergency Department of the University General Hospital of Patras, complaining of uterine contractions. This was her third pregnancy, completely unattended, due to her low socioeconomic status. The physical examination and cardiotocogram (CTG) evaluation revealed that the woman was in labor. A subsequent ultrasound examination of the fetus revealed marked macrocephaly and hydranencephaly (HC of 424 mm). Brainstem structures were present, thus the suspicion of holoprosencephaly was raised (Figure 1). Fetal biometry corresponded to 39 weeks, based on all estimated fetal weight parameters rather than HC alone. By family history, it emerged that she and her partner were second-degree relatives. Due to the apparent cephalopelvic disproportion, a cesarean section was decided.

The newborn immediately after delivery was admitted to the neonatal intensive care unit due to mild respiratory distress; otherwise, no major neurological deficits were present during a rough physical examination. The birth weight of 4510 g corresponded to the 97th centile. A more detailed examination of the newborn revealed mild distention of cranial sutures. Laboratory tests were normal. Screening for congenital infections (TORCH) and karyotype analysis were negative. A new brain MRI was performed, and the findings were primarily compatible with lobar holoprosencephaly with fusion of the lateral ventricles and significant dilatation of the ventricular system. In addition, a Dandy-Walker type dysplasia was identified with enlargement of the posterior cranial fossa, hypoplasia of the cerebellar vermis, and aplasia of the floor of the fourth ventricle.

The newborn was transferred to “Agia Sophia” Children’s Hospital for further evaluation and long-term management. During scrutinizing tests, a significantly low blood concentration of plasminogen was found, and thus the aforementioned defects in the central nervous system (CNS) development were ultimately attributed to congenital plasminogen deficiency. A ventriculoperitoneal catheter for drainage of the cerebrospinal fluid (CSF) was placed by a specialized pediatric neurosurgeon to treat the increased intracranial pressure. The infant remained alive at the time this manuscript was written.

## Discussion

Ventriculomegaly is a common fetal CNS anomaly with an incidence of 0.3-2 cases per 1000 pregnancies [1]. It is defined as an increased width of the lateral ventricles of the brain beyond 10mm. It is divided into mild (width 10-12 mm), moderate (12-15 mm), and severe (>15 mm). The term hydrocephalus refers to the combination of severe ventriculomegaly and increased CSF pressure within the ventricular system [2]. The prognosis of isolated mild/moderate ventriculomegaly is favorable, with an incidence of developmental delay of approximately 8%, while in severe cases, the prognosis is worse, with only 23.5% survival beyond the age of two years, especially when CNS defects coexist [3]. Worldwide, the prevalence of congenital hydrocephalus varies from less than 1 in 10000 live births in Denmark to 7 per 1000 in Egypt, while 78% of patients show neurological deficits for the rest of their lives [4,5]. Nowadays, more than 90% of ventriculomegaly and hydrocephalus cases are detected prenatally via ultrasound during the second trimester of pregnancy [4,6]. When routine fetal anatomy screening raises a suspicion, advanced neurosonography and MRI of the brain are recommended, given that in 20% of cases, MRI identifies additional CNS abnormalities [2,7].

Plasminogen, as a precursor to plasmin, is mainly crucial for fibrinolysis, maintaining vascular patency, and hemostatic balance. Beyond that, it plays vital roles in wound healing, tissue repair, and resolution of inflammation, even in bone remodeling. It achieves these multifaceted functions by binding to cell surface receptors, facilitating cell migration, and influencing immune processes. Hypoplasminogenemia is transmitted as an autosomal recessive trait [8], which is consistent with the increased incidence in blood-related offspring [8-11]. Homozygous congenital plasminogen deficiency is an extremely rare genetic disorder, with an incidence of 1 in 1,000,000. It potentially affects all mucous membranes of the body, leading to the formation of pseudomembranes. In 81% of patients, it affects the conjunctivae, causing xylem conjunctivitis. In 12% of cases, there is concomitant development of obstructive hydrocephalus, associated with severe forms of the disease [10, 12]. Dandy-Walker dysplasia occurs in only 5% of these cases [10]. Historically, the first recorded case of severe hypoplasminogenemia with xylem conjunctivitis and obstructive hydrocephalus was in 1994 and involved an 18-month-old girl from Turkey, while since then the total number of reported cases worldwide does not exceed 20 [10, 12]. CNS involvement in plasminogen deficiency is unusual because the blood-brain barrier restricts plasminogen entry, the CNS has alternative fibrin-clearance pathways, and typical fibrin-rich pseudomembranes form on mucosal, not neural, surfaces.

## Conclusions

In conclusion, mild ventriculomegaly is a non-rare finding on fetal anatomy screening with a favorable outcome, in comparison to fetal hydrocephalus. Furthermore, congenital plasminogen deficiency is an extremely rare cause of congenital hydrocephalus, but should be included in the differential diagnosis, especially in the absence of other more common causes and in offspring of blood relatives. Our case also highlights the need for proper and timely prenatal screening, as the management of such cases is challenging with a dubious prognosis.
